# Oxaliplatin-Related Hypersensitivity Reactions: A Single Institution Series and Literature Review

**DOI:** 10.3390/biomedicines10123275

**Published:** 2022-12-17

**Authors:** Francesca Barbin, Michele Ghidini, Alessandra Panichi, Gianluca Tomasello, Claudia Bareggi, Barbara Galassi, Nerina Denaro, Fiorella Ruatta, Carolina Cauchi, Maria Grazia Rossino, Ornella Garrone

**Affiliations:** Medical Oncology Fondazione IRCCS Ca’ Granda Ospedale Maggiore Policlinico, 20122 Milan, Italy

**Keywords:** oxaliplatin, hypersensitivity, solid tumors, chemotherapy

## Abstract

Oxaliplatin-based chemotherapy is extensively used for the treatment of gastrointestinal tumors and other malignancies. Oxaliplatin-related hypersensitivity reactions (HSRs) are common during antitumor treatment. Several studies have been conducted to identify predictive risk factors for oxaliplatin-related HSRs, but findings remain controversial. No definitive approach has been identified to reduce the risk of developing HSRs. The aim of this article is to provide an overview of oxaliplatin-related HSRs, and to report our institution’s experience. With our work, we reviewed available data from the literature and described our case series. A total of 153 patients were treated with oxaliplatin and 17 developed an HSR. On the whole, 70.6% of reactions were Grade 3, mostly with respiratory and cutaneous symptoms. Steroids and antihistamines were administered to reduce hypersensitivity symptoms and prevent further reactions. A stronger premedication and prolonged time of infusion resulted in milder reactions or absence of subsequent reactions. We did not find any clear predictive factor for the development of HSRs. Although it is not possible to cancel the risk of oxaliplatin-based HSRs, strategies to reduce the risk of occurrence could be stronger premedication and prolonged time of infusion.

## 1. Introduction

Oxaliplatin is a cytotoxic agent widely used for the treatment of gastrointestinal tumors and other malignancies [1]. It belongs to the group of alkylating agents, for which the mechanism of action is the interaction with DNA to form intrastrands and interstrand DNA crosslinks, thus affecting DNA pairing, replication and gene transcription, leading to cell death [2,3,4]. 

Oxaliplatin is associated with common adverse events such as peripheral neuropathy, vomiting, nausea, diarrhea, fatigue and cytopenias, but hypersensitivity reactions (HSRs) have also been described [2,3,5,6,7,8]. Drug-related toxicity frequently results in the interruption and/or delay of treatment administration, dose decrease and drug withdrawal, impeding progress toward cancer treatment. Once HSRs occur, the decision to re-administer the agent and to continue and complete the therapy is to be wisely considered [9]. Because of their unpredictable nature and severity, HRSs to antineoplastic agents frequently lead to the prospect of abandoning the treatment and switching to a another that can be less effective [10]. Oxaliplatin has demonstrated significant efficacy in the treatment of gastrointestinal tract cancers and other malignancies, and replacing it with another agent can negatively affect patients’ survival [2,11]. 

HSRs are unexpected and acute reactions that cannot be explained by the known toxicity profile of the drug and can occur during the first infusion or after repeated exposures [12,13]. Symptoms of HSRs usually manifest from some minutes after the start of the infusion to some hours after the end of the infusion, and their prompt detection is crucial for their management and the patients’ safety. Oxaliplatin-related HSRs can occur from the first administration; however, with an increased number of infusions the risk of reaction can increase accordingly [2,5,14,15]. They can be classified as mild, with symptoms such as palmar or facial flushing, rash, urticaria, itching and erythema, but they may progress to severe reactions with Grade 3–4 bronchospasm, laryngospasm, hypotension or anaphylaxis that is sometimes fatal [2,16,17]. The reported frequency of oxaliplatin-related HSRs has increased over time, due to the extensive use of this agent in several therapeutic settings [16]. Historically, the reported incidence of HSRs during oxaliplatin treatment was 1.8% [15], while the estimated actual incidence is 15–25% among all patients treated with oxaliplatin-based regimens [18]. The management of HSRs is an issue because drug interruption is usually required. Oxaliplatin rechallenge should be wisely considered, based on the severity of the reaction. However, it is frequently required to permanently discontinue the drug and change the treatment regimen. Several studies have been conducted to identify potential predictive risk factors for oxaliplatin-related HSRs, but available data in the literature are heterogeneous and findings remain controversial [15,19]. Because the management of these reactions and the adherence to the treatment program have important consequences on the patients’ quality of life and survival, it is critical to investigate the presence of potential predictive and risk factors for these types of reactions [12,19]. In addition, no definitive approach has been identified to reduce the risk of developing an HSR, since the pathophysiology of the reaction is still not clear. Strategies that have been proposed are the use of corticosteroids and antihistamines premedication and the reduction of the infusion rate and desensitization, but the reaction can still occur [2,3,8,18].

The aim of this article is to provide an overview of oxaliplatin-related HSRs, and to report the experience of our institution, in order to investigate possible predictors of oxaliplatin-related HSRs [19]. Based on studies found in the literature, this work provides updated data and information about oxaliplatin-related HSRs and their management, which is still an open issue in cancer treatment.

## 2. Materials and Methods

We performed a literature review on the PubMed database on October 2022. Search terms included: oxaliplatin, chemotherapy, cancer, hypersensitivity, adverse reactions, management and infusion-related reactions. Moreover, we compared results obtained from the literature with the case series of oxaliplatin-related HSR at our institution. We included patients who received at least one dose of oxaliplatin-based chemotherapy from September 2020 to September 2022. Data of patients, anonymized, were collected in a dedicated database. Variables analyzed were sex, age, primary tumor, purpose of treatment, treatment regimen, prior exposure to platinum salts, presence of pre-existing allergies, dose of oxaliplatin, type of premedication, total infusion course, blood count during first reaction, cycle of therapy during which reaction occurred, symptoms, severity, management of reaction, response to treatment, rechallenge (if performed), prophylaxis during rechallenge, subsequent reaction, symptoms, severity, management of reaction and response to treatment. HSRs were recorded and graded according to the National Cancer Institute—Common Terminology Criteria for Adverse Events (NCI-CTCAE, version 5.0). More in detail, allergic reaction/hypersensitivity is defined as a disorder characterized by an adverse local or general response from exposure to an allergen. Intervention is indicated based on grade of reaction: no intervention for Grade 1, oral intervention for Grade 2, intravenous and urgent intervention for Grade 3 and 4, respectively. Anaphylaxis is defined as a disorder characterized by an acute inflammatory reaction resulting from the release of histamine and histamine-like substances from mast cells, causing a hypersensitivity immune response. Clinically, it presents with breathing difficulty, dizziness, hypotension, cyanosis and loss of consciousness and may lead to death. Parenteral intervention and urgent intervention are indicated for Grade 3 and 4, respectively [20].

## 3. Results

From September 2020 to September 2022, 153 patients were treated with oxaliplatin at the Medical Oncology department of Fondazione IRCCS Ca’ Granda Ospedale Maggiore Policlinico and 17 (11%) of them experienced a hypersensitivity reaction (HSR). Demographic and baseline characteristics of patients are reported in Table 1.

Ten (58.8%) patients who experienced oxaliplatin-related HSRs were female and seven (41.2%) were male, with a median age of 69 years old (range 51–82).

**Figure 1:**
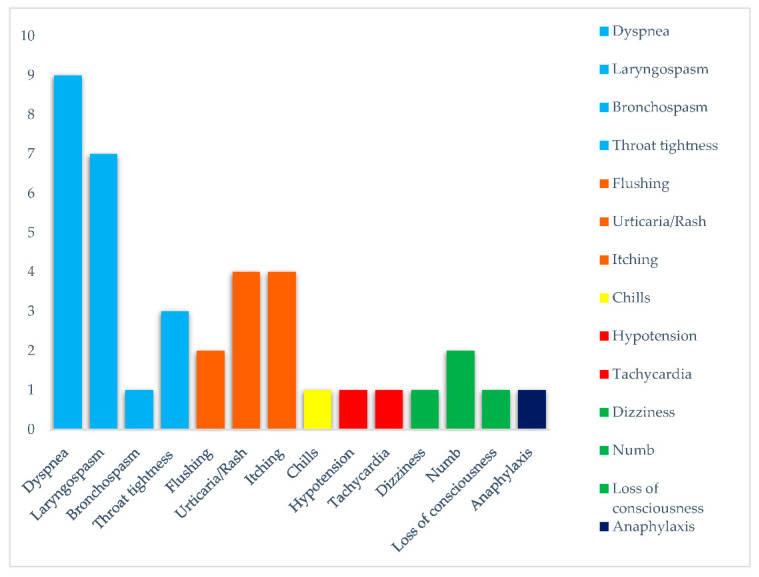
Symptoms of hypersensitivity to oxaliplatin.

Fourteen (82.4%) patients suffered from colorectal cancer, one (5.9%) from pancreatic cancer and two (11.7%) from biliary tract cancer. The purpose of treatment was palliative in 14 (82.4%) and adjuvant in 3 (17.6%) patients. Oxaliplatin was administered in combination with other drugs in the whole population: six (35.3%) patients were treated with XELOX (capecitabine and oxaliplatin), two (11.7%) patients with FOLFOX (folinic acid, 5-fluorouracil and oxaliplatin), two (11.7%) patients with XELOX and bevacizumab, one patient with FOLFOX and bevacizumab, two (11.7%) patients with FOLFOX and cetuximab, two (11.7%) patients with FOLFOX and panitumumab and two (11.7%) patients FOLFOXIRI (folinic acid, 5-fluorouracil, oxaliplatin and irinotecan) and bevacizumab. Concerning prior exposure to platinum salts, eight (47.1%) patients were previously treated. Only three (17.6%) patients had known allergies in their previous medical history. Patients received a median dose of 85 mg/m^2^ (60–130) of oxaliplatin. HSRs occurred after administration of a median of two (range 1–11) oxaliplatin infusions, and patients were treated with a median of six total cycles (range 1–13). The most frequent symptoms related to HSRs were respiratory: nine (52.9%) patients experienced Grade 3 dyspnea; seven (41.2%) patients experienced laryngospasm whose severity was Grade 2 for one reaction, Grade 3 for five reactions and Grade 4 for one reaction; one (5.9%) patient had Grade 4 bronchospasm; three (17.6%) patients reported throat tightness sensation with one case of Grade 2 and two of Grade 3 severity. Cutaneous symptoms reported were Grade 3 flushing in two (11.7%) patients, urticaria and rash in four (23.5%) patients, which were one Grade 1, one Grade 2 and two Grade 3 reactions; Grade 3 itching was reported in four (23.5%) patients. Only one patient experienced a Grade 4 anaphylactic reaction. No HSRs related deaths occurred. Other HSR-related symptoms are reported in Table 2 and Figure 1.

For all patients experiencing an HSR the infusion was immediately interrupted, and 14 (82.4%) patients received intravenous corticosteroids. Different doses of hydrocortisone were administered based on the severity and on the presence of one or more symptoms: 15 (88.2%) patients received i.v. hydrocortisone: 250 mg, 500 mg and 1 g in three, nine and three patients, respectively. Only five (29.4%) patients received intravenous antihistamines (10 mg chlorpheniramine) and three (17.6%) patients needed oxygen therapy to fully recover from symptoms. The only patient who experienced a Grade 4 anaphylactic reaction required corticosteroids (1 g hydrocortisone), antihistamines (10 mg chlorpheniramine), epinephrine (1 mg) and oxygen administration. Subsequent admittance to the emergency room was required to allow full recovery from the HSR. All except 1 of the 17 patients received premedication with dexamethasone, with a median dose of 12 mg (8–18.8); only 5 (29.4%) patients were premedicated with histamine blockers. Complete recovery from symptoms and the restart of the infusion of other chemotherapeutic drugs was seen in all except one patient after a median of 60 min (range: 30–80) from the onset of symptoms. The patient who was admitted to the emergency room was discharged 24 h later. We do not know the exact time to complete remission of symptoms. Overall, 13 (76.5%) patients received oxaliplatin rechallenge, regardless of the grade of the reaction that occurred (Grade 1–3). The strategy adopted to minimize the risk of a second reaction was the preventive administration of corticosteroids and antihistamines, and the prolongation of the time of infusion (60 min longer than the previous infusion). For one patient, oxaliplatin dose was also reduced. However, six (46.2%) patients experienced a second reaction: three (50.0%) patients reported cutaneous symptoms such as diffuse skin rash with itching and flushing, one (16.7%) patient experienced dyspnea and bronchospasm, one (16.7%) dyspnea and dysarthria, and one (16.7%) cutaneous, respiratory and neurological symptoms such as diffuse erythema, dyspnea, chills and mandibular dysesthesia. All reactions were Grade 3. The infusion was immediately interrupted, and corticosteroids were administered. Four (66.7%) patients received i.v. hydrocortisone 500 mg and one (16.7%) received hydrocortisone 250 mg. Only one (16.7%) patient was treated with paracetamol. None of them received antihistamines. The complete remission of symptoms was reached after a median of 60 min (range: 35–60). Two (33.3%) patients were rechallenged. A prolonged infusion rate was decided along with a stronger premedication starting from the day before the infusion with the oral administration of steroids and antihistamines for one of them. Both patients experienced a third reaction with milder symptoms: Grade 2 dyspnea and bronchospasm for the patient who received a more intense premedication, and diffuse skin rash for the other. The patient who experienced bronchospasm and dyspnea received intravenous infusion of hydrocortisone 1 g, with subsequent permanent discontinuation of oxaliplatin. For the patient who experienced cutaneous symptoms, treatment administration was interrupted and oxaliplatin was rechallenged a third time with premedication with steroids and antihistamines and prolongation of the infusion rate. No reaction occurred. Patients were retrospectively investigated for blood exams at baseline and at the time of the HSR’s onset, showing that at the time of the first reaction, eight (47.1%) patients had white blood cell (WBC) values below the lower normal limit (LNL), seven (41.2%) had monocytes count above upper normal limit (UNL) and three (17.6%) had an eosinophils count below the LNL. Furthermore, at the time of the second reaction, five (83.3%) of patients had a WBC count below the LNL, two (33.3%) had a monocyte count above the UNL and two (33.3%) had an eosinophils count below LNL. Concerning patients who did not experience any HSR, 123 out 136 patients (90.4%) received more than one oxaliplatin administration. Forty-five (35.6%) patients required oxaliplatin dose reduction (median 25%, range 10–50%) after a median of four cycles (range 2–10). Twenty (44.4%) patients completed all the planned chemotherapy cycles. A total of six (13.3%) patients were administered with a different premedication: 10 mg of chlorpheniramine were added before the infusion in four cases (66.7%), 250 mg of hydrocortisone was used in two (33.3%) patients, while one (25.0%) patient received and 500 mg of hydrocortisone.

Seventy-six (61.8%) patients did not reduce the oxaliplatin dose and 25 (32.9%) patients received the planned infusions. Among these latter patients, nine (36.0%) received additional premedication: six (66.7%) received 10 mg of chlorpheniramine, while four (44.4%) and two (22.2%) received 500 mg and 250 mg of hydrocortisone, respectively.

## 4. Discussion

Despite the use of oxaliplatin having increased over time, the pathophysiology of oxaliplatin-related HSRs is still not fully understood, and predictive factors of hypersensitivity have not been identified yet. The purpose of this research was to review the data available in the literature and to compare them with the more recent events occurred at our center.

Taking into consideration the literature available on oxaliplatin-related HSRs, we found articles and reviews from 2003 [21] to 2022 [6]. Among the total number of patients treated with oxaliplatin in every disease setting, the incidence rate of HSRs is very heterogeneous across different reports, with a median value of 15% (range 1.8–37.96). This percentage is in line with the data of our case series (11%) [22,23]. Park et al. [19] observed the presence of two peaks of occurrence for the first HSRs at the third and sixth cycle of treatment with oxaliplatin, while other authors individuate the eighth (range 3–10) as the median cycle of HSR occurrence [1,2,3,5,6,15,16,24,25,26,27,28]. At our center, we observed HSRs occurring after a median of two cycles (range 1–11), earlier compared to the literature data. There is no sufficient evidence supporting that both sex and age might influence the risk of HSR onset. Many reports [3,15,24] individuate an increased risk of HSR among female patients, while others found an increased incidence in male patients [4,6,19,25,29]. Median age of HSR onset was 56.2 years in historical data and 69 years in our series.

Regarding the severity of HSR, we found a greater number of Grade 3–4 reactions with respect to the literature data, but NCI-CTCAE versions used to grade and identify the reactions are not aligned. Moreover, Grade 1 and 2 reactions that occurred after the end of the infusion may have been underestimated and not reported. The low incidence of Grade 1–2 reactions might depend on the fact that most patients were premedicated with intravenous corticosteroids and selective 5-HT3 antagonists before the infusion and it might also reflect the fact that milder infusion reactions were excluded from reporting [5]. Symptoms observed were mainly respiratory (70.6%), with dyspnea and laryngospasm being the most frequently reported symptoms. Cutaneous reactions occurred in 23.5% of patients with urticaria, itching and flushing. These data are consistent with reports described by Maindrault-Goebel et al. [16]. Data are consistent regarding the management of HSRs, with the immediate interruption of the infusion and the administration of symptomatic therapy such as corticosteroids and antihistamines to reduce and stop symptoms. The duration of the reaction and the complete recovery from symptoms is also congruent with the literature data, with a rapid and total remission of symptoms. The choice to administer an oxaliplatin rechallenge in patients experiencing HSRs depends on the physician’s choice, considering patient’s condition, severity of the previous reaction, response to symptomatic treatment and intention to treat the patient [30]. In our series, we rechallenged patients who experienced Grade 3 reactions even though the literature data does not support it. However, patients rechallenged with oxaliplatin were strongly premedicated and infusions were prolonged as per the literature’s advice. No desensitization protocol was adopted at our center.

HSRs can be classified as “Type B” reactions, based on the European Medicines Agency (EMA) definition of adverse drug reactions (ADRs): non-dose related, unpredictable and usually not related to drug’s mechanism of action and usually resolving when treatment is terminated. Type B reactions are divided into immune-mediated reactions and non-immune reactions. Gell and Combs defined a more specific classification of these reactions, recognizing four hypersensitivity states. Type I reactions are immunoglobulin E (IgE)-mediated reactions, such as anaphylaxis; Type II reactions are antibody-mediated reactions such as thrombocytopenia, hemolytic anemia and blood transfusion reactions; Type III reactions are immune-complex-mediated hypersensitivity reactions, such as vasculitis and serum sickness; Type IV reactions are delayed T cell-mediated reactions, such as erythema multiforme, toxic epidermal necrolysis, allergic contact dermatitis. According to the onset of symptoms, the European Network for Drug Allergy (ENDA) has categorized HSRs into two types of reactions: immediate, with symptoms onset within 1–6 h from the drug exposure, typically IgE-mediated, and non-immediate, with symptoms onset at any time, from 1 h after the initial exposure to many days after the end of the exposure. These reactions are usually delayed T cell-mediated allergic reactions [31,32,33,34]. It is known that platinum agents, together with their direct cytotoxic effects, also impact the immune system and can lead to immune cell activation [35]. The mechanism and pathophysiology of HSRs is still unclear, but in 7 (41.2%) out of 17 patients who experienced an HSR, a monocyte count above the UNL was registered. We could hypothesize that the reaction is a delayed drug hypersensitivity reaction. In fact, monocytes–macrophages and dendritic cells are antigen-presenting cells, components of the innate immune system, and they are activated by foreign antigens, as with platinum salts [8,36]. Their activation stimulates cytokines and other co-stimulating molecules that provide signals to activate resting T cells. T cells themself also produce monocyte activation [37,38,39]. Furthermore, we did not find high levels of eosinophil as described by Okayama et al. [29], but we found three (17.6%) patients with lower levels of eosinophils. Eosinophil count seems not to be a predictive factor for HSR, agreeing with Sohn et al. [25]. WBC count could be decreased because of the known toxicity of chemotherapeutic agents, and this is not considered a predictive or risk factor for developing an HSR. These data could be explored because they are opposite to values founded by Seki et al. [40], who suggests that low monocyte count and neutrophil levels are a predictive marker of oxaliplatin related HSRs. Moreover, they found that serum lactate dehydrogenase (LDH) levels are a predictive marker of oxaliplatin-induced GSRs. In our series, only one patient had low neutrophil counts and the LDH value was not available, therefore these data could not be investigated and compared.

Our study has some limitations compared to literature studies cited above: first of all, it is a retrospective series collected at single center. Our analysis has taken into consideration only 2 years of treatment, compared to a median of 4, 5 years of treatment with oxaliplatin emerged in the literature review. Moreover, because of the retrospective nature of the study, some data were missing: LDH was not available and could not be compared to literature data, and baseline blood exams of patients who did not experience an HSR were not performed at our center. Moreover, Grade 1–2 reactions that did not require the interruption of the infusion might have been underestimated and underreported, such as reactions that occurred some hours after the end of the infusion, when the patient was already discharged, or mild self-limiting reactions.

We could focus on a homogeneous population and used reproducible strategies in order to identify and manage oxaliplatin-related HSRs. Patients who experienced a Grade 3 HSR were able to receive a safe drug rechallenge with a more intense premedication not only in the context of the infusion but also in the days preceding the treatment. This strategy might be developed and explored to minimize the risk of drug withdrawal, especially in a treatment context where there are not so many alternative options available.

This work is based on studies, reviews and case reports already present in the literature, but available data is heterogeneous and controversial. At our institution, we do not use a desensitization protocol. Grade 3 HSRs were safely rechallenged with the strategy of intense premedication, not only during the infusion but also in the days preceding the treatment administration. Furthermore, since HSRs are unpredictable and unexpected reactions as per their definition, no randomized trials can be performed to investigate their causes and nature. The analyses of more recent and updated data available through retrospective studies are an important instrument to characterize and improve HSR management.

## 5. Conclusions

Oxaliplatin-related HSRs are still an open issue in cancer treatment. Their incidence has been seen increasing over the last years in parallel with the extensive use of this drug. However, we still cannot find reliable predictive and risk factors of their occurrence. The only potential reported risk factor that has been found to be consistent through the literature data available is the repetitive exposure to oxaliplatin, even though reactions can occur even at the very first administrations.

Premedication with dexamethasone seems to be a protective factor for the development of mild reactions (Grade 1–2), together with the preventive administration of antihistamines before the infusion in order to minimize the risk of triggering an allergic reaction. Another strategy that could be taken into consideration is to strengthen the premedication and prolong the infusion rate.

Our case series leaves some issues open: with our retrospective and single-center experience we noticed that premedication with dexamethasone and antihistamines starting from the first administrations of oxaliplatin seems to be a good protective strategy. Prolongation of the infusion time and a stronger premedication could be another protective and preventive factor, but infusion rate, which doses and when the right moment to introduce it is are not clear. At last, it is not clear if there is any clear detectable predictive factor in blood exam values. There is still unclarity about the possible correlation between HSRs pathophysiology and blood exam counts, and data available are heterogeneous, but it should be explored.

It is clear that a percentage of HSRs will always occur among all patients treated with oxaliplatin, but it would be advisable to identify predictive and risk factors in order to minimize their occurrence and their impact on patients’ quality of life, treatment and survival.

## Figures and Tables

**Table 1:** Demographic and baseline characteristics of patients.

	Study Population	Pts with HSRs
	n = 153 ^1^	n = 17
	(100%)	(11%)
Female	83 (54.3)	10 (58.8)
Male	70 (45.7)	7 (41.2)
Age, years	70 (27–87)	69 (51–82)
(median, range)		
**Diagnosis**		
Colorectal cancer	108 (70.6)	14 (82.4)
Pancreatic cancer	12 (7.8)	1 (5.9)
Biliary tract cancer	6 (3.9)	2 (11.7)
Stomach cancer	26 (17.0)	0 (0)
Esophagus cancer	1 (0.7)	0 (0)
**Chemotherapy regimen**		
FOLFOX	21 (13.7)	2 (11.7)
XELOX	71 (46.4)	6 (35.3)
FOLFOX + bevacizumab	17 (11.0)	1 (5.9)
FOLFOX + cetuximab	2 (1.3)	2 (11.7)
FOLFOX + panitumumab	13 (8.5)	2 (11.7)
FOLFOX + trastuzumab	1 (0.7)	0 (0)
FLOT	6 (3.9)	0 (0)
FOLFIRINOX	3 (2.0)	0 (0)
FOLFOXIRI + bevacizumab	4 (2.6)	2 (11.7)
XELOX + bevacizumab	13 (8.5)	2 (11.7)
XELOX + trastuzumab	2 (1.3)	0 (0)
**Purpose of treatment**		
Adjuvant	65 (42.5)	3 (17.6)
Palliative	88 (57.5)	14 (82.4)
**Prior exposure to platinum salts ^2^**		
Yes	30 (19.6)	8 (47.1)
No	123 (80.4)	9 (52.9)
**Number of infusions**		
Median (range)	5 (1–12)	6 (1–11)
**Premedication ^3^**		
Steroids (8 mg)	13 (8.5)	2 (11.7)
Steroids (12 mg)	137 (89.5)	13 (76.5)
Steroids (18.8 mg)	0 (0)	1 (5.9)
Antihistamines (10 mg)	37 (24.2)	5 (29.4)
Antiemetics	153 (100)	17 (100)
**History of allergic diseases**		
Yes	22 (14.4)	3 (17.6)
No	131 (85.6)	14 (82.4)

^1^ One hundred and fifty-three patients received oxaliplatin infusions from September 2020 to September 2022. It includes patients that experienced an oxaliplatin-related hypersensitivity reaction in the same period. ^2^ Some patients were pretreated with cisplatin-based regimen. ^3^ Patients received dexamethasone and hydrocortisone, chlorpheniramine and ondansetron, 8 mg or palonosetron 300, 5 mg as premedication before the infusion. Abbreviations: FLOT—5-fluorouracil, oxaliplatin, docetaxel; FOLFIRINOX—folinic acid, 5-fluorouracil, oxaliplatin and irinotecan; FOLFOX—folinic acid, 5-fluorouracil and oxaliplatin; FOLFOXIRI—folinic acid, 5-fluorouracil, oxaliplatin and irinotecan; HSR—hypersensitivity reactions; Pts—patients; XELOX—capecitabine and oxaliplatin.

**Table 2:** Hypersensitivity reactions reported in the study population.

Total Reactions	n = 17 (%)
**Severity ^1^**	
Grade 1	1 (5.9)
Grade 2	3 (17.6)
Grade 3	12 (70.6)
Grade 4	1 (5.9)
Grade 5	0 (0)
**Cycle number of event** **(median, range)**	2 (1–11)
**Premedication**	
Steroids	17 (100)
Antihistamines	5 (29.4)
Antiemetics	17 (100)
**Symptoms**	
Respiratory	
Dyspnea	9 (52.9)
Laryngospasm	7 (41.2)
Bronchospasm	1 (5.9)
Throat tightness	3 (17.6)
Cutaneous	
Flushing	2 (11.7)
Urticaria/rash	4 (23.5)
Itching	4 (23.5)
General	
Chills	1 (5.9)
Cardiovascular	
Hypotension	1 (5.9)
Tachycardia	1 (5.9)
Neurological	
Dizziness	1 (5.9)
Numb	2 (11.7)
Loss of consciousness	1 (5.9)
Anaphylaxis	1 (5.9)
**Management of reaction**	
Infusion interruption	17 (100)
Steroids administration	14 (82.4)
Antihistamines administration	5 (29.4)
Oxygen administration	3 (17.6)
Saline solution administration	3 (17.6)
Epinephrine administration	1 (5.9)
**Rechallenge**	
Yes	13 (76.5)
No	4 (23.5)
**Subsequent Reaction**	
Yes	6 (46.2)
No	7 (53.8)

^1^ Note: from *Common Terminology Criteria for Adverse Events* (v.5.0), by the National Cancer Institute—Cancer Therapy Evaluation Program, 2017. Retrieved from https://ctep.cancer.gov/protocoldevelopment/electronic_applications/docs/ctcae_v5_quick_reference_5x7.pdf (accessed on 3 October 2022).

## Data Availability

The data presented in this study are available on request from the corresponding author. The data are not publicly available due to privacy restrictions.
